# Predictive value of ApoB/ApoA-I for recurrence within 1 year after first incident stroke

**DOI:** 10.3389/fneur.2023.1308442

**Published:** 2024-01-11

**Authors:** Jie Lin, Zhihua Si, Aihua Wang

**Affiliations:** ^1^Department of Neurology, Shandong Provincial Qianfoshan Hospital, Shandong University, Shandong Institute of Neuroimmunology, Shandong Key Laboratory of Rheumatic Disease and Translational Medicine, Shandong, China; ^2^Department of Neurology, The First Affiliated Hospital of Shandong First Medical University and Shandong Provincial Qianfoshan Hospital, Shandong Institute of Neuroimmunology, Shandong Key Laboratory of Rheumatic Disease and Translational Medicine, Shandong, China

**Keywords:** ApoB/ApoA-I, stroke recurrence, acute ischemic stroke, low density lipid cholesterol, high density lipid-cholesterol

## Abstract

**Background:**

ApoB/ApoA-I ratio is a reliable indicator of cholesterol balance, particularly in the prediction of ischemic events risk. The aim of this study was to investigate the prognostic value of ApoB/ApoA-I for stroke recurrence within 1 year after the first incident.

**Methods:**

We retrospectively included patients who were first diagnosed with acute (<7 days after onset) ischemic stroke. Blood samples were collected on admission, and serum ApoB and ApoA-I concentrations were measured. We analyzed the relationship between ApoB/ApoA-I ratio and ischemic stroke recurrence within 1 year.

**Results:**

A total of 722 patients with acute ischemic stroke were included, of whom 102 experienced stroke recurrence within 1 year, with a recurrence rate of 14.1%. Serum ApoB/ApoA-I concentrations on admission were higher in patients with stroke recurrence at 1 year compared with those with a good prognosis (*P* < 0.001). The Kaplan-Meier survival curve revealed a significant difference in cumulative stroke recurrence rates across ApoB/ApoA-I tertiles (log-rank *P*-value < 0.001). A positive correlation between the ApoB/ApoA-I ratio and the risk of stroke recurrence within 1 year was demonstrated using Cox regression analysis, which remained significant after adjusting for traditional risk factors [hazard ratio (HR) 4.007, 95% confidence interval (CI) 1.661–9.666]. This relationship was particularly strong in patients with LAA stroke (HR 4.955, 95% CI 1.591–15.434). Subgroup analysis further revealed that a high ApoB/ApoA-I ratio was strongly associated with stroke recurrence regardless of whether patients had high or low LDL-C levels.

**Discussion:**

ApoB/ApoA-I ratio, measured during the acute phase of the first stroke, was positively correlated with the risk of stroke recurrence within 1 year.

## Background

The proportion of human deaths and disabilities caused by stroke remains unacceptably high worldwide. In high-income countries, the incidence, mortality, and disability rates of stroke have significantly declined due to aggressive primary and secondary prevention efforts for stroke. However, people in low- and middle-income countries have continued to bear a significant economic and health burden as a result of stroke over the past few decades due to population explosions, aging populations, and growing incidences of intervenable risk factors for stroke (1, 2). Acute ischemic stroke (AIS) which is prevalent in China's cerebrovascular disease is at an increasing stage, and after the onset of AIS, ~17.7% of the population will experience a recurrence of cerebral infarction (3). Aggressive treatment and prevention efforts for stroke are of great value in reducing the economic and health burden on a nation and its people.

Stroke is a complex condition with a multitude of underlying pathogenic mechanisms, among which large-artery atherosclerotic (LAA) stroke stands as the most prevalent subtype (4). Dyslipidemia is a key and adjustable risk factor for atherosclerosis and has long been a subject of great interest. Among them, the connection between low-density lipoprotein cholesterol (LDL-C), a traditional lipid marker, and atherosclerotic disease has been extensively studied. Unfortunately, according to available research, clinical interventions targeting solely the traditional indicator of LDL-C have failed to curb atherosclerotic lesion progression, even when LDL-C levels are reduced to within the target range (5, 6). Therefore, exploring the relationship between new lipid markers and atherosclerosis risk is of great interest to researchers. Low-density lipoprotein (LDL), very low-density lipoprotein (VLDL), intermediate density lipoprotein (IDL), lipoprotein a, and celiac particles all contain a single apolipoprotein B (ApoB) molecule, and quantification of ApoB provides a direct measure of the number of atherogenic particles in the plasma (7). One of the primary components of high-density lipoprotein (HDL) is ApoA-I, which maintains the structural integrity of HDL particles, a finding that was recognized as early as the 1870s (8). The ApoB/ApoA-I ratio indicates the balance between LDL-C and HDL-C, an imbalance of which accelerates the development of atherosclerosis and thereby increases the risk of ischemic stroke (9, 10). The ApoB test is standardized and relatively inexpensive, and fasting is not required for its measurement (11). However, there is a lack of research into the correlation between ApoB/ApoA1 and stroke recurrence after the first episode. The purpose of this study was to clarify the prognostic value of ApoB/ApoA1 for stroke recurrence and alleviate future cerebrovascular disease.

## Materials and methods

### Participants

Clinical data of 783 patients with first diagnosis of acute (<7 days after onset) ischemic stroke and relatively complete hospitalization data were retrospectively collected from the Department of Neurology, The First Affiliated Hospital of Shandong First Medical University, between September 2020 and June 2022. The study was approved by the ethical review committee of the First Affiliated Hospital of Shandong First Medical University [Reference No. (S402)], and informed consent was obtained from the patients and their families. All methods were performed in accordance with the relevant guidelines and regulations. Inclusion criteria were as follows: (1) first diagnosis of acute (<7 days after onset) ischemic stroke; (2) complete prior history, laboratory tests for lipid indexes, and imaging data; (3) acute infarction was diagnosed with reference to the China Acute Ischemic Stroke Diagnosis and Treatment Guidelines 2018, and all diagnoses were confirmed by cranial MRI examination (12). Exclusion criteria were as follows: (1) age of onset < 18 years; (2) patients who underwent thrombolysis or vascular intervention because thrombolysis and vascular interventions have a significant prognostic impact on patients with large-artery atherosclerosis stroke and are an important confounding factor for this study; (3) those with serious systemic comorbidities before hospitalization, such as severe pneumonia, hepatic insufficiency, renal failure, respiratory failure, and heart failure; (4) patients with stroke caused by coagulation disorders, blood composition alterations, vasculitis of varying origins, vascular malformations, and unknown reasons; (5) patients had taken statin lipid-lowering drugs 1 month prior to hospitalization.

### Data collection

All study patients had morning fasting venous blood drawn within 24 h of admission, and laboratory indices were measured including triglyceride (TG), total cholesterol (TC), high-density lipoprotein cholesterol (HDL-C), low-density lipoprotein cholesterol (LDL-C), apolipoprotein B, and apolipoprotein A-I. General clinical information of patients was collected by electronic medical records, including history of hypertension, diabetes mellitus, smoking, and alcohol drinking; and large-artery atherosclerosis (LAA) stroke, cardioembolic (CE) stroke, and small-vessel occlusive (SVO) stroke were evaluated using the Trial of Org 10172 in Acute Stroke Treatment (TOAST) classification (13), with the latter two collectively referred to as non-large-artery atherosclerotic stroke.

### Outcome measures

Patients were followed up by telephone at the 3rd and 12th months after discharge for a total of 1 year, beginning with the time of discharge and ending with ischemic stroke recurrence. The follow-up included (1) the occurrence of stroke recurrence and diagnosis of acute cerebral infarction by professionals or institutions within 1 year after discharge, with neurological impairment caused by cerebral hemorrhage, intracranial tumor, cerebral cell edema, brain herniation resulting in tissue displacement, post-infarction hemorrhage, etc. and excluded (2) duration from discharge to recurrent ischemic cerebrovascular events; (3) medication compliance: whether oral medications including antiplatelet drugs, lipid-lowering drugs, hypoglycemic drugs, and antihypertensive drugs were taken as prescribed by the discharge physician; if medications were interrupted, the reasons for discontinuation were to be investigated. If the medication is discontinued for more than 1 month without any reason, it is regarded as poor adherence to medication.

### Statistical analysis

All data were statistically analyzed using SPSS software version 25.0 and R4.1.1 (R Foundation). Based on the follow-up outcomes, the study subjects were divided into recurrence and non-recurrence groups. Demographic characteristics, previous history, and laboratory tests were compared between the two groups, using different statistical methods to compare the demographic characteristics, previous history, and laboratory tests of the two groups. Measurement data were first tested for normality, and they were expressed as mean ± standard deviation (X ± SD) if they obeyed normal distribution; Student's *t*-test was used for comparison between two groups; if they were not normally distributed, they were expressed as M(P25, P75), and Mann–Whitney *U*-test was used. Count data were displayed as percentages (%), using the χ2 test, with Kaplan–Meier curves used to estimate cumulative stroke recurrence rates for ApoB/ApoA-I categories (dichotomized by median). The restricted cubic spline function with four nodes (5th, 35th, 65th, and 95th percentiles) was used to explore the potential non-linear relationship between serum ApoB/ApoA-I ratio and stroke recurrence. Univariate and multivariate Cox regression analyses were used to examine the relationship between ApoB/ApoA-I and stroke recurrence. The following factors were adjusted for in the multivariate Cox regression: demographic variables (age and sex) and established vascular risk factors (hypertension, diabetes mellitus, dyslipidemia, active smoking, and medication compliance), regardless of their significance. The results were shown as adjusted hazard ratios (HRs) and their 95% confidence intervals (CIs). To further assess the predictive value of ApoB/ApoA-I for ischemic stroke recurrence, the area under the receiver operating characteristic curve (AUROC) was calculated for the models with and without ApoB/ApoA-I.

To investigate the potential heterogeneity of the relationship between ApoB/ApoA-I and stroke recurrence in subgroups, we performed stratified analyses. The stratification was based on age (cutoff at 60 years), sex, history of hypertension, history of diabetes mellitus, LDL cholesterol (cutoff at 2.65 mmol/L), and TOAST classification. Interaction analysis by stratification factors in multivariate Cox regression was conducted to assess the effect of ApoB/ApoA-I on outcomes. All *P*-values were two-tailed with a significance level of 0.05.

## Results

We screened a total of 783 patients with a first diagnosis of acute cerebral infarction who were hospitalized within 7 days after the development of symptoms of neurological impairment and excluded 13 patients with other serious systemic diseases at the time of admission. During follow-up, we excluded 21 patients who were lost to follow-up, 12 patients who died of non-cerebrovascular diseases, and 15 patients who developed neurological impairment due to cerebral hemorrhage, intracranial tumor, post-infarction hemorrhage, etc. Ultimately, 722 patients with acute cerebral infarction were enrolled, including 299 patients with large-artery atherosclerotic stroke, 265 with small-vessel occlusive stroke, and 158 with cardioembolic stroke. Among them, 102 patients had stroke recurrence within 1 year, with a recurrence rate of 14.1%. The serum ApoB/ApoA-I concentrations on admission were significantly higher in patients with stroke recurrence at 1 year compared to those with good prognosis (median 1.01 mmol/L vs. 0.84 mmol/L, *P* < 0.001). The proportion of patients with ApoB/ApoA-I > median was significantly higher in the recurrence group (18.3 vs. 9.9%, *P* < 0.001) compared to the non-recurrence group. Additionally, patients with stroke recurrence had higher plasma LDL-C levels, higher ApoA-I and ApoB concentrations, higher incidence of hypertension, higher incidence of diabetes mellitus, and poorer medication compliance compared to patients without stroke recurrence. The above results are shown in Table 1.

**Table 1:** Baseline characteristics of patients with and without stroke recurrence.

**Characteristics^a^**	**Stroke recurrence**		***X^2^/Z***	***P*-value**
	**No (*****n*** = **620)**	**Yes (*****n*** = **102)**		
Male (*n*)	394	71	1.403	0.236
Age (year)	64	66	−1.672	0.095
Hypertension (*n*)	373	72	4.027	0.045
Diabetes (*n*)	160	39	6.777	0.009
Smoking (*n*)	207	42	2.352	0.125
Alcohol drinking (*n*)	132	26	0.888	0.346
Poor medication adherence (*n*)	88	30	11.893	<0.001
TG (mmol/L)	1.27 (0.96, 1.79)	1.31 (0.89, 1.90)	−0.454	0.650
TC (mmol/L)	4.61 (4.06, 5.31)	4.80 (4.12, 5.63)	−1.399	0.162
LDL-C (mmol/L)	2.62 (2.11, 3.20)	2.80 (2.28, 3.36)	−1.966	0.049
HDL-C (mmol/L)	1.10 (0.94, 1.25)	1.04 (0.89, 1.18)	−1.944	0.052
Apolipoprotein A-I (g/L)	0.93 (0.82, 1.04)	0.84 (0.75, 0.96)	−4.088	<0.001
Apolipoprotein B (g/L)	0.80 (0.68, 0.98)	0.86 (0.72, 1.05)	−2.064	0.039
Apolipoprotein B/Apolipoprotein A-I	0.84 (0.72, 1.07)	1.01 (0.79, 1.27)	−3.722	<0.001
Apolipoprotein B/Apolipoprotein A-I category			10.488	0.001
<median (*n*)	320	35		
≥median (*n*)	300	67		
TOAST			2.150	0.143
LAA stroke (*n*)	250	49		
No-LAA stroke (*n*)	370	53		

TG, triglyceride; TC, total cholesterol; LDL-C, low-density lipoprotein cholesterol; HDL-C, high-density lipoprotein cholesterol; TOAST, Trial of Org 10172 in Acute Stroke Treatment; LAA, large-artery atherosclerosis; No-LAA stroke includes cardioembolic stroke and small-vessel occlusive stroke.

**Figure 1:**
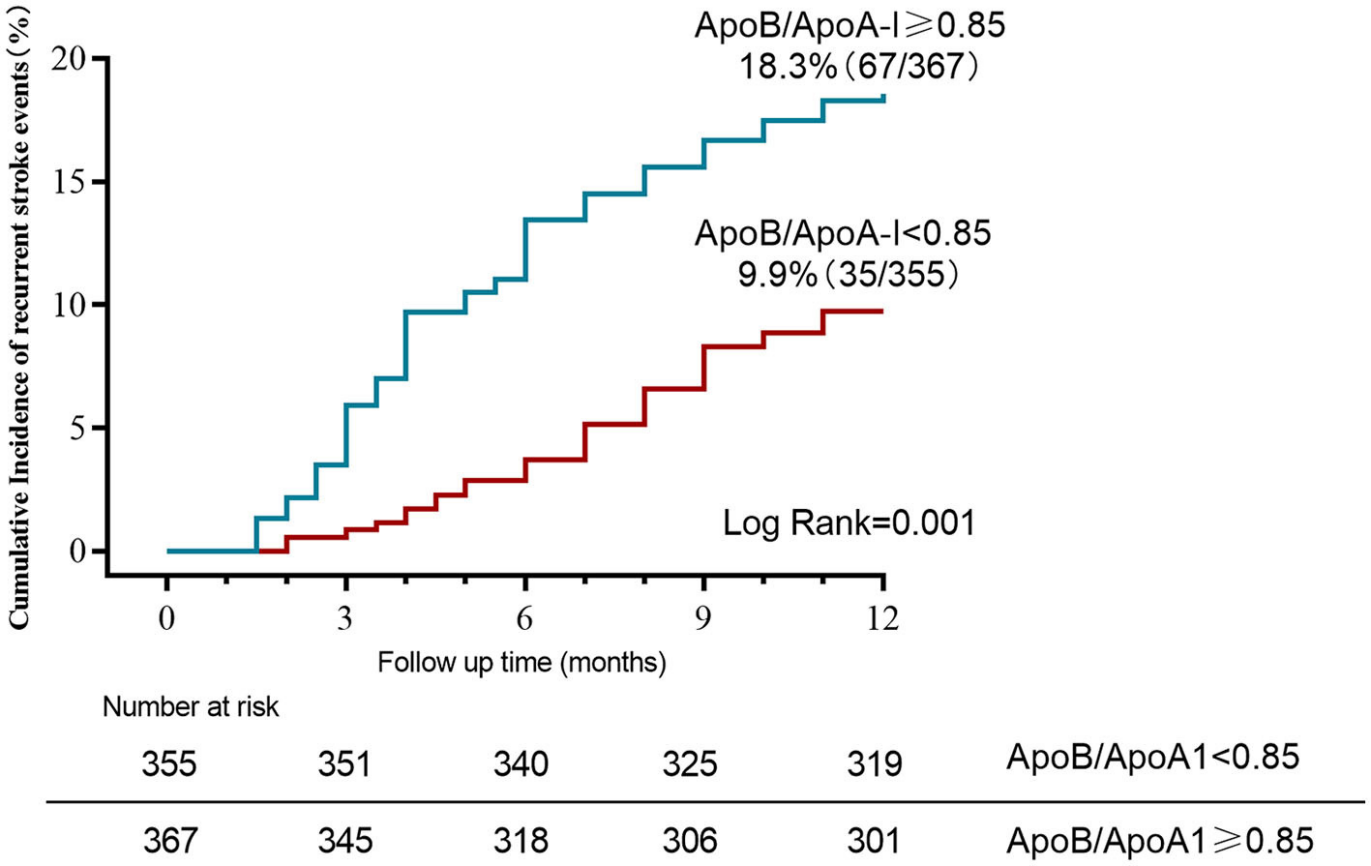
Kaplan–Meier curves estimate cumulative stroke recurrence rates for ApoB/ApoA-I categories (dichotomized by median) during 12-month follow-up.

When tested as a linear variable, the ApoB/ApoA-I ratio was higher in younger patients, current or former smokers, and those with a history of hypertension, high levels of LDL-C and TG, low levels of HDL-C, and large-artery atherosclerosis (LAA) strokes (Table 2). Dividing acute-phase serum concentrations of ApoB/ApoA-I into two groups, the cumulative stroke recurrence rate at the end of the 1-year follow-up was 18.3% for ApoB/ApoA-I > median and 9.9% for ApoB/ApoA-I < median, with significantly different cumulative stroke recurrence rates between the two groups (log-rank *P*-value = 0.001; Figure 1).

**Table 2:** ApoB/ApoA-I by patient characteristics.

**Characteristics**	**No**.	**Apolipoprotein B/Apolipoprotein A-I**	***Z***	***P*-value**
**Sex**			−1.623	0.105
Male	466	0.87 (0.74, 1.13)		
Female	257	0.85 (0.70, 1.07)		
**Age, year**			−2.007	0.045
<60	265	0.91 (0.77, 1.13)		
≥60	457	0.84 (0.72, 1.08)		
**History of hypertension**			−2.771	0.006
No	277	0.83 (0.72, 1.03)		
Yes	445	0.90 (0.74, 1.14)		
**History of diabetes**			−1.737	0.082
No	523	0.85(0.73, 1.07)		
Yes	199	0.90(0.73, 1.18)		
**Smoking**			−2.970	0.003
No	473	0.84 (0.71, 1.07)		
Yes	249	0.92 (0.78, 1.16)		
**Alcohol drinking**			−0.761	0.446
No	564	0.85 (0.73, 1.08)		
Yes	158	0.89 (0.73, 1.17)		
**TG category**			−6.602	<0.001
<median (1.28 mmol/L)	362	0.83 (0.68, 1.00)		
≥median (1.28 mmol/L)	360	0.94 (0.79, 1.19)		
**LDL-C category**			−13.705	<0.001
<median (2.65 mmol/L)	362	0.78 (0.65, 0.87)		
≥median (2.65 mmol/L)	360	1.05 (0.85, 1.23)		
**HDL-C category**			−3.779	<0.001
<median (1.09 mmol/L)	361	0.92 (0.77, 1.15)		
≥median (1.09 mmol/L)	361	0.83 (0.69, 1.04)		
**Toast**			−2.813	0.005
LAA	299	0.84 (0.71, 1.07)		
No-LAA	423	0.91 (0.75, 1.15)		

TG, triglyceride; TC, total cholesterol; LDL-C, low-density lipoprotein cholesterol; HDL-C, high-density lipoprotein cholesterol; TOAST, Trial of Org 10172 in Acute Stroke Treatment; LAA, large-artery atherosclerosis; No-LAA includes CE and SVO.

**Figure 2:**
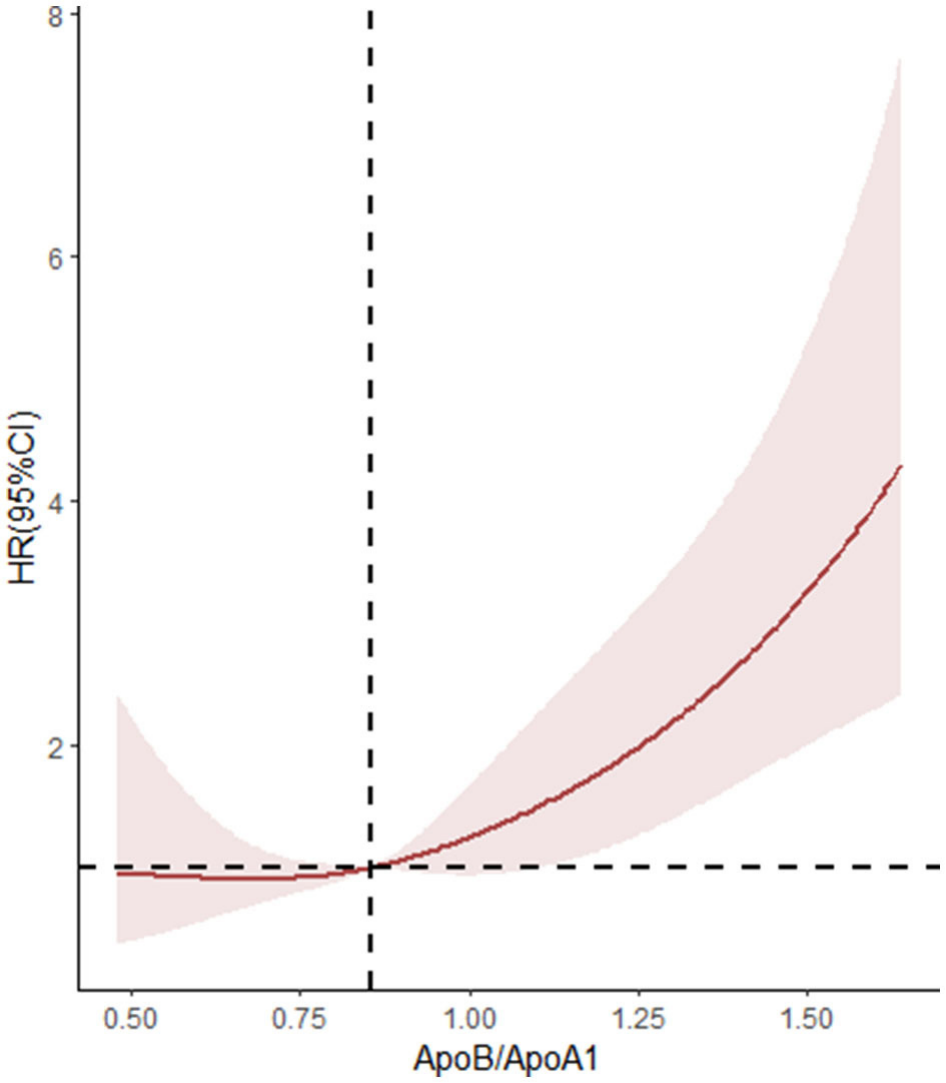
Non-linear associations between stroke recurrence and ApoB/ApoA-I ratio. HR where the revalue for the ApoB/ApoA-I ratio is 0.853. *P*-overall < 0.001, *P*-non-linear = 0.408.

The relationship between Apo B/Apo A-I and stroke recurrence was analyzed using univariate and multivariate Cox regression models, the results of which are shown in Table 3 and Supplementary Tables 1, 2. The ApoB/ApoA-I ratio, as well as age, diabetes, and poor medication adherence, were independently associated with 1-year stroke recurrence. In univariate Cox regression, the risk of stroke recurrence in patients with ApoB/ApoA-I above the median was nearly twice as high as in patients below the median (HR 1.975, 95% CI 1.312–2.973, *P* < 0.001). However, this association attenuated after mutual adjustment (HR 1.636, 95% CI 0.979–2.732, *P* = 0.060). In multivariate Cox regression, the ApoB/ApoA-I ratio as a continuous variable was an independent risk factor for stroke recurrence (HR 4.007, 95% CI 1.661–9.666, *P* < 0.001). The restricted cubic spline plot did not reveal a non-linear relationship between serum ApoB/ApoA-I and 1-year stroke recurrence (*P* = 0.402 for non-linear trend, Figure 2).

**Table 3:** Univariate and multivariate cox regression analyses for the association of ApoB/ApoA-I with stroke recurrence.

**Characteristics**	**Univariate analysis**						**Multivariate analysis** ^**a**^					
	β	**SE**	**Wald** χ^2^	***P*** **-value**	**HR**	**95%** ***CI***	β	**SE**	**Wald** χ^2^	***P*** **-value**	**HR**	**95%** ***CI***
ApoB/ApoA-I category	0.681	0.209	10.650	0.001	1.975	1.312~2.973	0.492	0.262	3.533	0.060	1.636	0.979–2.732
ApoB/ApoA-I	1.551	0.321	23.276	<0.001	4.717	2.512~8.857	1.388	0.449	9.543	0.002	4.007	1.661–9.666

a Adjusted for risk factors included age, sex, diabetes, hypertension, medication compliance, smoking, triglycerides, low-density lipoprotein cholesterol, and high-density lipoprotein cholesterol.

Subgroup analysis showed that ApoB/ApoA-I was independently associated with stroke recurrence regardless of the age of patients, the presence of hypertension, and the level of LDL-C. Additionally, ApoB/ApoA-I was significantly correlated with stroke recurrence in the no-diabetes and LAA subgroups. However, the hazard ratios of ApoB/ApoA-I were not significant in the subgroups of female individuals, history of diabetes, and non-LAA stroke (Table 4). The interaction analysis showed that stratified subgroups (including LDL cholesterol) did not have an interaction effect with ApoB/ApoA-I on stroke recurrence. The analysis of ROCs for stroke recurrence is shown in the Supplementary Table 3. The addition of the ApoB/ApoA-I category to the model including age, sex, and other risk factors increased the AUC from 0.688 to 0.708 and the sensitivity from 0.627 to 0.755 but decreased the specificity from 0.716 to 0.602.

**Table 4:** Subgroup analysis of odd ratios of ApoB/ApoA-I for stroke recurrence.

	**HR**	**95% CI**	***P*-value**	***P* interaction**
**Sex**				0.548
Male (*n* = 466)	4.713	1.778–12.493	0.002	
Female (*n* = 257)	2.279	0.360–14.444	0.382	
**Age**				0.241
<60 years (*n* = 265)	7.472	1.754–31.840	0.007	
≥60 years (*n* = 457)	3.170	1.198–8.383	0.020	
**Hypertension**				0.781
Yes (*n* = 445)	3.180	1.171–8.637	0.023	
No (*n* = 277)	5.778	1.396–23.918	0.016	
**Diabetes**				0.670
Yes (*n* = 199)	1.789	0.383–8.365	0.460	
No (*n* = 523)	7.213	2.485–20.935	<0.001	
**LDL-C**				0.456
<median (*n* = 362)	5.478	1.014–29.588	0.048	
≥median (*n* = 360)	3.436	1.366–8.642	0.009	
**TOAST**				0.638
LAA stroke (*n* = 299)	4.955	1.591–15.434	0.006	
Non-LAA stroke (*n* = 423)	2.921	0.692–12.333	0.145	

Hazard ratios were adjusted for age, sex, diabetes, hypertension, medication compliance, smoking, triglycerides, low-density lipoprotein cholesterol, and high-density lipoprotein cholesterol, except for the stratified variable.

TOAST, Trial of Org 10172 in Acute Stroke Treatment; LDL-C, low-density lipoprotein cholesterol; LAA, stroke large-artery atherosclerosis; Non-LAA includes CE and SVO.

## Discussion

Based on current studies, we found that elevated ApoB/ApoA-I levels were associated with stroke recurrence within 1 year after the first acute cerebral infarction, and this association remained statistically significant after adjusting for potential risk factors. Additionally, patients with acute cerebral infarction who were older than 60 years, suffering from hypertensive disorders, chronic smokers, and dyslipidemia, had higher levels of ApoB/ApoA-I. The addition of ApoB/ApoA-I to the traditional risk model improved the predictive power of the model, implying that ApoB/ApoA-I is a good indicator in predicting stroke recurrence.

A significant positive correlation was found between dyslipidemia, including elevated levels of LDL-C and TC, and the incidence of ischemic stroke (14). However, some studies have shown that ApoB/ApoA-I is a better indicator of cholesterol balance than traditional lipid markers, especially in predicting the risk of ischemic events such as coronary heart disease, myocardial infarction, and ischemic stroke (15–17). Donnell et al. in an international multicenter case–control study found that non-HDL-C/HDL-C ratios and ApoB/ApoA1 ratios were significantly correlated with ischemic stroke. Multivariate regression analysis adjusting for multiple ischemic stroke risk factors showed that the ApoB/ApoA1 ratio had a higher predictive value for ischemic stroke (OR: 2.33, 95% CI 1.80–3.00) than the non-HDL-C/HDL-C ratio (OR: 1.47, 95% CI 1.17–1.86) (18). Kostapanos et al. investigated the predictive value of ApoB/ApoA1 in relation to acute ischemic non-embolic stroke in the elderly. A total of 163 patients aged 70 years were included, and an elevated ApoB/ApoA1 ratio was shown to be an independent predictor of ischemic stroke in people aged 70 years and older in a multivariate regression analysis (19). In this study, we recruited a cohort with a first diagnosis of ischemic stroke and found that ApoB/ApoA-I was an independent risk factor for stroke recurrence and showed good predictive value.

The prognosis and risk factors for stroke recurrence vary among different ischemic stroke subtypes. Patients with recurrent multiple lacunar infarcts are more susceptible to cognitive dysfunction and have a higher prevalence of hypertension compared to other subtypes (20). The prevalence of atrial fibrillation and early recurrence rates are higher in patients with cardioembolic stroke compared to other subtypes, playing an important role in predicting in-hospital mortality (21, 22). Plasma LDL-c levels are now recognized as an independent risk factor for LAA, while the relationship with other stroke types is unclear (21). Therefore, it is necessary to explore the relationship between ApoB/ApoA-I and different stroke subtypes. It has been suggested that there is an association between the ApoB/ApoA-I ratio and intracranial atherosclerotic stenosis (23, 24). Kalani et al. found no significant difference in ApoB and ApoA-I levels in populations with different ischemic stroke subtypes (25). The evidence provided by a Mendelian randomization study supports the suggestion that apolipoprotein B is a major feature of the etiologic basis of ischemic stroke, particularly in large-artery and small-vessel stroke (26). In the subgroup analysis of the present study, the ApoB/ApoA-I ratio was found to be independently associated with LAA stroke recurrence compared to non-LAA strokes. This phenomenon may be explained by the pathogenic mechanisms of apolipoprotein B and apolipoprotein A-I. The pathogenesis of atherosclerosis is complex and involves subendothelial retention of lipoproteins, aggregation, and oxidation of the retained lipoproteins, endothelial damage and inflammation, macrophage chemotaxis and foam cell formation, and smooth muscle cell migration and alterations. Because each atherogenic particle has only one ApoB molecule, the amount of ApoB more accurately reflects the number of atherogenic particles in the circulation (27). The concentration of ApoB in the arterial lumen is the main determinant of the entry of ApoB particles into the arterial wall and their retention under the endothelium. The more ApoB particles in the arterial lumen, the more lipoproteins are retained in the arterial wall. After retention in the arterial intima, the aggregated ApoB particles are phagocytosed by macrophages and converted to foam cells (28). LDL contains ApoB-100, and ApoB causes LDL to be recognized and trapped by LDL receptors on arterial wall cells, leading to impaired endothelial lipid peroxidation and endothelium-dependent vasodilation in the artery, increasing vasoconstriction and promoting the development of ischemic vascular disease (29). ApoA-I is the major apolipoprotein associated with HDL, and the main mechanism by which HDL is protective against atherosclerosis is reverse cholesterol transport. ApoA-I, as a major structural component, is intimately involved in the synthesis and reverse transport of HDL, with anti-atherosclerotic properties that inhibit oxidative modification of lipoproteins and protect endothelial cell function (30). The pathogenic mechanisms of ApoB and ApoA-I suggest that the ratio represents a balance between pro- and anti-atherosclerotic, pro- and anti-inflammatory, and thrombotic and antithrombotic lipoprotein particles and has great clinical application in stroke risk assessment and targeted therapy. Due to the fact that the pathophysiology, prognosis, and clinical features of small-vessel ischemic strokes are different from other stroke subtypes (31), the association of ApoB/ApoA-I with small-vessel stroke is uncertain. The pathophysiology of small-vessel stroke is highly diverse and can lead to lacunar infarction due to vascular occlusion, disrupted cerebral autoregulation, or augmented vascular permeability (32). Additionally, small-vessel occlusion may be attributed to a thrombogenic mechanism resembling that of large-artery atherosclerosis. A future line of research on the discussed topic would be to study the relationship and relevance of the predictive value of ApoB/ApoA-I for recurrence within 1 year after the first incident stroke in ischemic small-vessel disease vs. other ischemic stroke subtypes.

According to the subgroup analyses in this study, we also found that a high ApoB/ApoA-I ratio was strongly associated with an increased risk of stroke recurrence regardless of whether the patients had high or low LDL-C levels. Similar to the results of a study on ApoB and coronary artery disease, Kim et al. found in 14,205 patients without cardiovascular disease, including 2,773 patients with mild coronary artery disease, followed for 5 years, indicating that high plasma ApoB levels were closely related to the prevalence and progression of coronary artery calcium and were not affected by LDL-C levels (33). The abovementioned study confirmed that ApoB is a predictor of cardiovascular and cerebrovascular disease within the target range of LDL-C. Therefore, simultaneous measurement of traditional lipid markers (including TG, TC, LDL-C, and HDL-C), ApoB, and ApoA-I is more conducive to risk assessment of cardiovascular and cerebrovascular diseases. However, due to the limited sample size, the results of other subgroup analyses must be interpreted with prudence. Future multicenter and large sample size studies are needed to further confirm these findings.

Currently, dyslipidemia remains one of the major challenges in the management of patients with cerebrovascular disease. The reduction of plasma LDL levels through inhibition of hydroxymethylglutaryl coenzyme A reductase (statin therapy) is central to the guidelines for the treatment of dyslipidemia (12), but there is still a significant residual risk in patients with stroke after statin therapy. The next most promising alternative therapy for the treatment of atherosclerosis is the direct reduction of ApoB levels. Lomitapide and mipomersen sodium were recently approved by the US Food and Drug Administration for the adjuvant treatment of patients aged ≥18 and ≥12 years, respectively, with pure-sibling familial hypercholesterolemia (HoFH) (34, 35). Lomitapide is an oral inhibitor of microsomal triglyceride transport protein (MTP), which reduces hepatic production of apoB-containing lipoproteins by inhibiting MTP (36). Mipomersen is a second-generation antisense nucleotide targeting ApoB mRNA that reduces ApoB synthesis, resulting in reduced production of atherosclerotic lipoproteins (37). The current drugs commonly used in clinical practice only raise HDL-C by 35%, prompting researchers to search for new compounds to raise HDL (38). One approach is to directly administer HDL or its components, so investigating ApoA-I mimetic compounds or recombinant HDL particles is a promising treatment strategy, and a representative drug is ApoA-I Milan (39, 40). ApoA-I Milan exhibits multiple beneficial effects on lipid metabolism and cardiovascular health. Specifically, it enhances cholesterol efflux from cells, thereby promoting its elimination from the body. Furthermore, ApoA-I Milan exerts a protective effect on LDL particles, shielding them from oxidative damage that can lead to the development of atherosclerotic plaques. Additionally, this protein induces lipolysis, a process that breaks down fats, which may contribute to weight loss. Finally, ApoA-I Milan has been observed to reduce inflammatory markers and plaque vulnerability, suggesting a potential role in mitigating the risk of cardiovascular events (41, 42).

## Limitations

This study has several limitations. First, the clinical endpoints at follow-up were assessed by telephone interview rather than clinical consultation, which increases the risk of missing potentially relevant endpoints, especially TIA. Second, the follow-up and documentation of the issue of patient adherence to medication were not detailed enough. We only recorded the presence or absence of discontinuation, which would have been more complete if the type of discontinuation had been included in the prediction model. Third, as a single-center retrospective analysis, our study included only the patients tested with ApoB/ApoA-I, and selection bias definitely exists. The severity of stroke is related to the risk of stroke recurrence. However, this study did not collect patients' National Institute of Health stroke scale (NIHSS) scores at admission, which is one of the limitations. Finally, this study only analyzed the relationship between stroke recurrence and ApoB/ApoA-I during the first year in patients with first acute ischemic stroke but did not analyze the correlation at other time points (at 3 months, 6 months, etc.) between ApoB/ApoA-I and stroke recurrence; otherwise, the findings would have been more complete. The strength of this study lies in its pioneering discovery of a positive correlation between ApoB/ApoA-I levels and the risk of recurrence of first ischemic stroke, a correlation that is particularly significant in LAA stroke patients. These findings suggest that ApoB and ApoA-I may serve as novel therapeutic targets for stroke patients in the future.

## Conclusion

The findings of the current study suggest an association between the ratio of ApoB to ApoA-I measured during the acute phase of the first stroke and the risk of stroke recurrence within 1 year. After adjusting for traditional prognostic factors, this association remained significant, particularly in the LAA stroke subgroup and independently of LDL-C levels. Elevated plasma levels of ApoB/ApoA-I are an independent risk factor for stroke recurrence within 1 year of acute ischemic stroke. Targeted lowering of both LDL-C levels and ApoB/ApoA-I may provide additional benefits for patients with cerebral infarction. Future studies are needed to determine whether ApoB/ApoA-I can be used as a target for secondary prevention of cerebrovascular disease.

## Data availability statement

The raw data supporting the conclusions of this article will be made available by the authors, without undue reservation.

## Ethics statement

The studies involving humans were approved by the Ethics Review Committee of the First Affiliated Hospital of Shandong First Medical University. The studies were conducted in accordance with the local legislation and institutional requirements. The participants provided their written informed consent to participate in this study.

## Author contributions

JL: Data curation, Investigation, Methodology, Software, Writing – original draft, Writing – review & editing. ZS: Funding acquisition, Writing – review & editing. AW: Funding acquisition, Writing – review & editing.
