# Supplementary material for: Predictive value of ApoB/ApoA-I for recurrence within 1 year after first incident stroke
Source: Front Neurol. 2024 Jan 11;14:1308442. doi: 10.3389/fneur.2023.1308442 (PMC10808791; doi:10.3389/fneur.2023.1308442)
Supplement: Supplementary file 1 [file Table_1.DOCX]

| Characteristics | Univariate analysis | | | | | |
| --- | --- | --- | --- | --- | --- | --- |
|  | β | SE | Wald χ^2^ | *P-value* | OR | 95% *CI* |
| ApoB/ApoA-I category | 0.681 | 0.209 | 10.650 | 0.001 | 1.975 | 1.312~2.973 |
| ApoB/ApoA-I | 1.551 | 0.321 | 23.276 | <0.001 | 4.717 | 2.512~8.857 |
| Sex(male=1) | -0.246 | 0.215 | 1.311 | 0.252 | 0.782 | 0.513-1.192 |
| Age | 0.017 | 0.010 | 2.996 | 0.083 | 1.017 | 0.998-1.036 |
| Hypertension | 0.446 | 0.217 | 4.215 | 0.040 | 1.562 | 1.020-2.392 |
| Diabetes | 0.534 | 0.204 | 6.872 | 0.009 | 1.706 | 1.144-2.544 |
| Smoking | 0.300 | 0.201 | 2.222 | 0.136 | 1.350 | 0.910-2.002 |
| Alcohol consumption | 0.196 | 0.227 | 0.743 | 0.389 | 1.216 | 0.779-1.899 |
| Medication compliance | 0.811 | 0.217 | 13.927 | <0.001 | 2.250 | 1.470-3.446 |
| TG | 0.058 | 0.091 | 0.409 | 0.522 | 1.060 | 0.886-1.268 |
| TC | 0.145 | 0.094 | 2.357 | 0.125 | 1.156 | 0.961-1.391 |
| LDL-C | 0.241 | 0.123 | 3.839 | 0.050 | 1.272 | 1.000-1.619 |
| HDL-C | -0.786 | 0.429 | 3.363 | 0.067 | 0.456 | 0.197-1.056 |
| Apolipoprotein E | 0.006 | 0.008 | 0.619 | 0.432 | 1.006 | 0.991-1.022 |
| Apolipoprotein A-I | -2.330 | 0.622 | 14.033 | <0.001 | 0.097 | 0.029-0.329 |
| Apolipoprotein B | 1.022 | 0.438 | 5.452 | 0.020 | 2.778 | 1.178-6.551 |

eTable1 Univariate Cox logistic regression analysis of variables with stroke recurrence

Abbreviations: TG triglycerides, TC total cholesterol, LDL-C low-density lipoprotein cholesterol, HDL-C high-density lipoprotein cholesterol

eTable2 Multivariate Cox logistic regression analysis of variables with stroke recurrence

| Characteristics | ApoB/ApoA-I category | | | | | | ApoB/ApoA-I levels | | | | | |
| --- | --- | --- | --- | --- | --- | --- | --- | --- | --- | --- | --- | --- |
|  | β | SE | Wald χ^2^ | *P-value* | OR | 95% *CI* | β | SE | Wald χ^2^ | *P-value* | OR | 95% *CI* |
| Sex(male=1) | -0.302 | 0.258 | 1.369 | 0.242 | 0.739 | 0.446-1.226 | -2.15 | 0.261 | 0.681 | 0.409 | 0.806 | 0.484-1.344 |
| Age | 0.027 | 0.011 | 6.498 | 0.011 | 1.028 | 1.006-1.049 | 0.027 | 0.011 | 6.293 | 0.012 | 1.027 | 1.006-1.049 |
| Hypertension | 0.439 | 0.222 | 3.921 | 0.048 | 1.552 | 1.004-2.398 | 0.411 | 0.222 | 3.418 | 0.064 | 1.509 | 0.976-2.334 |
| Diabetes | 0.594 | 0.211 | 7.902 | 0.005 | 1.811 | 1.197-2.739 | 0.543 | 0.211 | 6.621 | 0.010 | 1.721 | 1.138-2.604 |
| Smoking | 0.185 | 0.234 | 0.626 | 0.429 | 1.203 | 0.761-1.904 | 0.184 | 0.233 | 0.626 | 0.429 | 1.202 | 0.762-1.899 |
| Medication compliance | 0.889 | 0.224 | 15.779 | <0.001 | 2.433 | 1.569-3.773 | 0.844 | 0.225 | 14.116 | <0.001 | 2.326 | 1.489-3.614 |
| TG | -0.18 | 0.108 | 0.027 | 0.870 | 0.983 | 0.796-1.213 | -0.021 | 0.111 | 0.037 | 0.847 | 0.979 | 0.788-1.216 |
| LDL-C | 0.112 | 0.161 | 0.485 | 0.486 | 1.119 | 0.816-1.533 | -0.077 | 0.177 | 0.188 | 0.664 | 0.926 | 0.655-1.310 |
| HDL-C | -0.327 | 0.493 | 0.441 | 0.507 | 0.721 | 0.274-1.894 | -0.032 | 0.498 | 0.004 | 0.949 | 0.968 | 0.365-2.568 |
| ApoB/ApoA-I≥median (0.85) | 0.492 | 0.262 | 3.533 | 0.060 | 1.636 | 0.979~2.732 | / | / | / | / | / | / |
| ApoB/ApoA-I | / | / | / | / | / | / | 1.388 | 0.449 | 9.543 | 0.002 | 4.007 | 1.661~9.666 |

Abbreviations: TG triglycerides, LDL-C low-density lipoprotein cholesterol, HDL-C high-density lipoprotein cholesterol


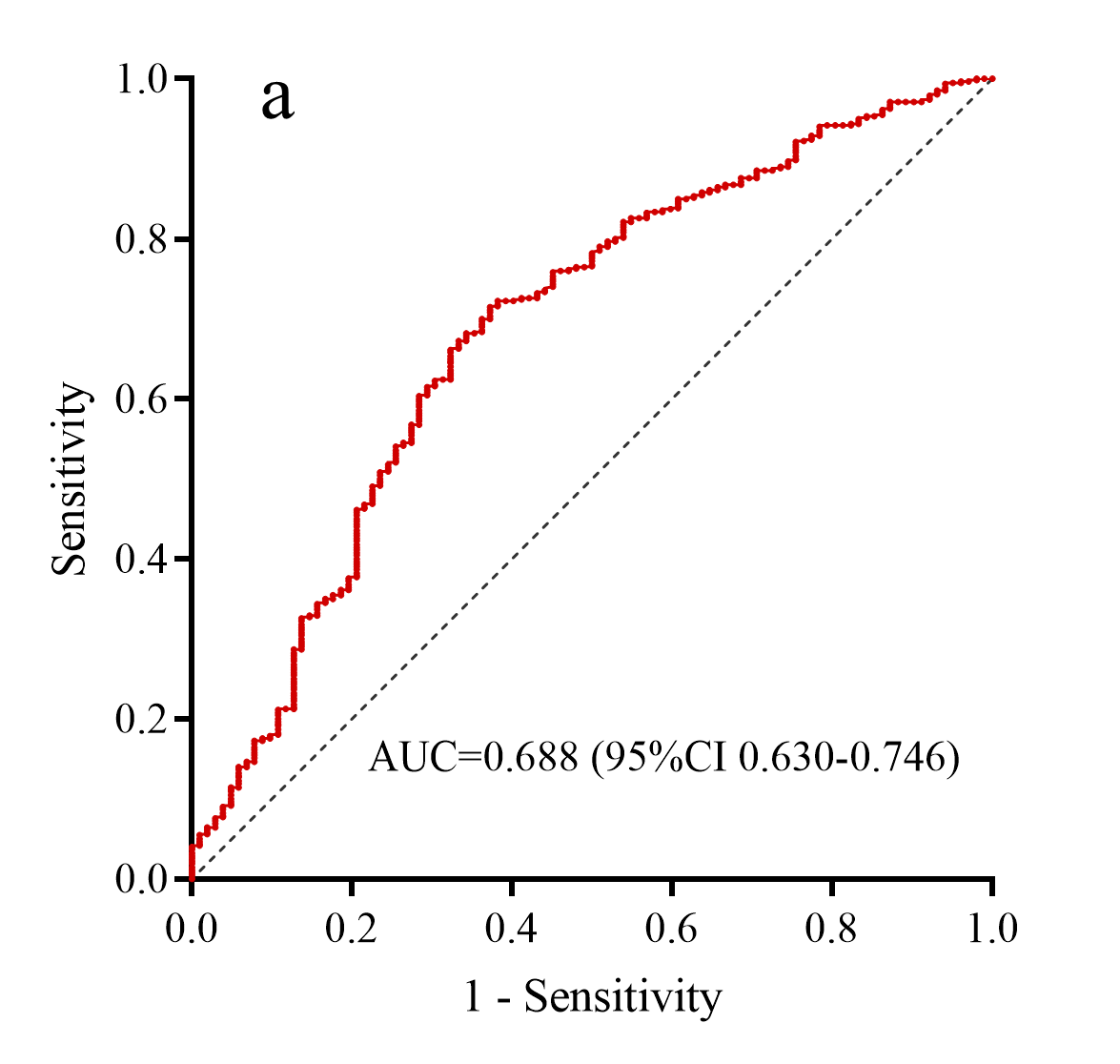

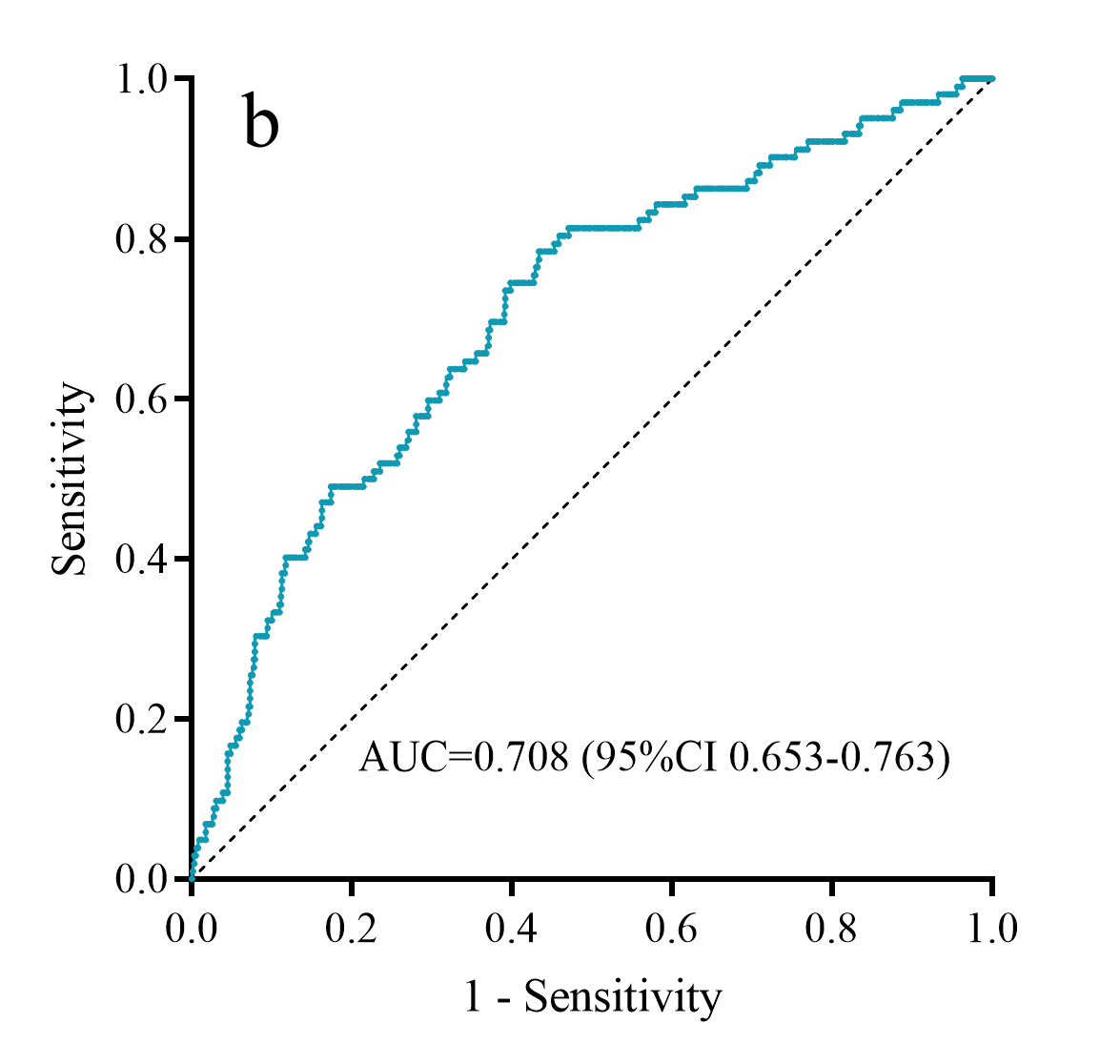


eFig.1 Receiver operator curves (ROC) of predicting stroke recurrence for **a model** containing age, sex and vascular risk factors, **b model** a plus ApoB/ApoA-I ratio. Abbreviations: AUC, area under curve; CI, confidence interval.

|  | AUC (95% *CI*) | Sensitivity | Specificity | Youden Index |
| --- | --- | --- | --- | --- |
| a model | 0.688(0.603, 0.746) | 0.627 | 0.716 | 0.343 |
| b model | 0.708 (0.653, 0.763) | 0.755 | 0.602 | 0.357 |

eTable3 Receiver operator curves (ROC) of predicting stroke recurrence for **a model** and **b model**
